# Development and evaluation of a novel radioiodinated vesamicol analog as a sigma receptor imaging agent

**DOI:** 10.1186/2191-219X-2-54

**Published:** 2012-09-28

**Authors:** Kazuma Ogawa, Hiroya Kanbara, Kazuhiro Shiba, Yoji Kitamura, Takashi Kozaka, Tatsuto Kiwada, Akira Odani

**Affiliations:** 1 Division of Pharmaceutical Sciences, Graduate School of Medical Sciences, Kanazawa University, Kakuma-machi, Kanazawa 920-1192, Japan; 2 Advanced Science Research Center, Kanazawa University, 13-1 Takara-machi, Kanazawa, 920-8640, Japan

**Keywords:** Sigma receptor, Imaging, Cancer

## Abstract

**Background:**

Sigma receptors are highly expressed in human tumors and should be appropriate targets for developing tumor imaging agents. Previously, we synthesized a vesamicol analog, (+)-2-[4-(4-iodophenyl)piperidino]cyclohexanol ((+)-*p*IV), with a high affinity for sigma receptors and prepared radioiodinated (+)-*p*IV. As a result, (+)-[^125^I]*p*IV showed high tumor uptake in biodistribution experiments. However, the accumulation of radioactivity in normal tissues, such as the liver, was high. We supposed that some parts of the accumulation of (+)-*p*IV in the liver should be because of its high lipophilicity, and prepared and evaluated a more hydrophilic radiolabeled vesamicol analog, (+)-4-[1-(2-hydroxycyclohexyl)piperidine-4-yl]-2-iodophenol ((+)-IV-OH).

**Methods:**

(+)-[^125^I]IV-OH was prepared by the chloramine T method from the precursor. The partition coefficient of (+)-[^125^I]IV-OH was measured. Biodistribution experiments were performed by intravenous administration of a mixed solution of (+)-[^125^I]IV-OH and (+)-[^131^I]*p*IV into DU-145 tumor-bearing mice. Blocking studies were performed by intravenous injection of (+)-[^125^I]IV-OH mixed with an excess amount of ligand into DU-145 tumor-bearing mice.

**Results:**

The hydrophilicity of (+)-[^125^I]IV-OH was much higher than that of (+)-[^125^I]*p*IV. In biodistribution experiments, (+)-[^125^I]IV-OH and (+)-[^131^I]*p*IV showed high uptake in tumor tissues at 10-min post-injection. Although (+)-[^131^I]*p*IV tended to be retained in most tissues, (+)-[^125^I]IV-OH was cleared from most tissues. In the liver, the radioactivity level of (+)-[^125^I]IV-OH was significantly lower at all time points compared to those of (+)-[^131^I]*p*IV. In the blocking studies, co-injection of an excess amount of sigma ligands resulted in significant decreases of tumor/blood uptake ratios after injection of (+)-[^125^I]IV-OH.

**Conclusions:**

The results indicate that radioiodinated (+)-IV-OH holds a potential as a sigma receptor imaging agent.

## Background

Originally, sigma receptors were proposed as a new subtype of opioid receptors in 1976 [1]. At present, it is known that sigma receptors possess specific drug selectivity characteristics and unique properties as different types of receptors from the opioid receptors. It has been reported that there are at least two subtypes of sigma receptors, designated sigma-1 and sigma-2 [2]. The sigma-1 receptor subtype has been cloned from various tissues and species [3]. The human sigma-1 receptor is a transmembrane protein of 223 amino acids [4], which is located on the outer cell membrane and the endoplasmic reticulum. Recently, the sigma-2 receptor subtype, whose gene remains to be cloned, has been identified as being progesterone receptor membrane component 1 [5]. In the central nervous system, sigma receptors have been shown to be involved in the regulation of neurotransmitter release, modulation of neurotransmitter receptor function, learning and memory processes, and regulation of movement and posture [6]. Sigma receptor ligands could be candidate drugs as neuroprotective agents after a stroke or head trauma [7], as antidepressant agents [8], as anti-amnesic agents [9], as analgesic agents [10], for alcohol abuse [11], and so on.

At the same time, it has been reported that both sigma receptor subtypes are highly expressed in a variety of human tumors such as prostate cancer, breast cancer, malignant melanoma, renal carcinomas, colon carcinomas, glioma, neuroblastoma, small cell lung carcinoma, and non-small cell lung carcinoma [12-14]. The high expression of sigma receptors in tumors suggests that they are appropriate targets for developing tumor-imaging agents. Furthermore, sigma receptors should be potential biomarkers of tumor proliferation because they are highly expressed in rapidly proliferating cells and are downregulated when cells become quiescent [15-17]. Meanwhile, sigma receptor ligands also could be candidate drugs for cancer therapy because some ligands have been reported to affect cell growth and apoptosis [18,19]. Thus, imaging sigma receptors might have potential for predicting prognosis and early diagnosis of the therapeutic effects of the drugs by determining the expression level of the sigma receptor and drug development by determination of receptor occupancy. For now, it was also reported that radiolabeled sigma ligands should be useful for monitoring the early effects of chemotherapy before morphologic changes are observed [20].

**Figure 1:**
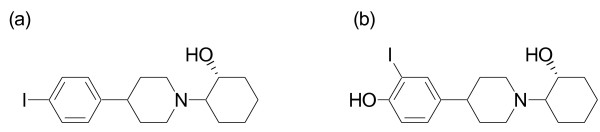
**Chemical structures of (a) (+)-*p*IV and (b) (+)-IV-OH.**

Previously, we have developed several vesamicol analogs with iodine into the 4-phenylpiperidine moiety as sigma receptor imaging agents and determined the binding affinities for the sigma receptors of the vesamicol analogs [21,22]. In these vesamicol analogs, the (+)-enantiomer of 2-[4-(4-iodophenyl)piperidino]cyclohexanol ((+)-*p*IV, Figure 1a) showed the highest affinities for the receptors [22]. Thus, to evaluate the potential of radioiodinated (+)-*p*IV for tumor imaging, biodistribution experiments of (+)-^125^I]*p*IV using tumor-bearing mice were performed. As a result, (+)-^125^I]*p*IV showed high uptake and long residence in the tumor. High tumor to blood and muscle ratios were achieved because the radioactivity levels of blood and muscle were low. However, the accumulations of radioactivity in normal tissues, such as the liver and kidney, were high [23].

We supposed that some parts of the accumulation of (+)-*p*IV in normal tissues, especially in the liver, should be because of its high lipophilicity. In this study, we designed and synthesized a new vesamicol derivative, (+)-4-[1-(2-hydroxycyclohexyl)piperidine-4-yl]-2-iodophenol ((+)-IV-OH, Figure 1b), which is a more hydrophilic compound compared with (+)-*p*IV by introduction of a hydroxyl group to the benzene ring of vesamicol. Preparation of radioiodinated (+)-IV-OH was performed. Usefulness of the new radioiodinated compound as a sigma receptor imaging agent was evaluated *in vitro* and *in vivo*.

## Methods

### Materials

Proton nuclear magnetic resonance spectra were recorded on a JEOL JNM-ECS400 spectrometer (JEOL Ltd., Tokyo, Japan), and the chemical shifts were reported in parts per million downfield from an internal tetramethylsilane standard. Electrospray ionization mass spectra were obtained with an LCQ mass spectrometer system (Thermo Fisher Scientific, Waltham, MA, USA). Optical rotations were measured with a SEPA-300 high-sensitive polarimeter (HORIBA, Kyoto, Japan). [^3^H]1,3-Di-tolylguanidine ([^3^H]DTG) (1.1 TBq/mmol), [^3^H]pentazocine (1.0 TBq/mmol), [^125^I]sodium iodide (644 GBq/mg), and [^131^I]sodium iodide (185 GBq/mg) were purchased from PerkinElmer (Waltham, MA, USA). Thin layer chromatography (TLC) analyses were performed with silica plates (Art 5553, Merck, Darmstadt, Germany). SA4503 was kindly supplied by M's Science (Kobe, Japan). DTG, pentazocine, and haloperidol were purchased from Sigma Chemical (St. Louis, MO, USA). Other reagents were of reagent grade and used as received.

### Preparation of (+)-enantiomer of 4-[1-(2-hydroxycyclohexyl)piperidine-4-yl]-2-iodophenol ((+)-IV-OH, 7)

(+)-Enantiomer of compound 5 (274 mg, 1 mmol), which was prepared using a method described previously [24,25], was dissolved in 1 mL of 1-M HCl and then NaNO_2_ (172 mg, 2.5 mmol) in 0.5 mL of water was added dropwise to the solution. The reaction solution was stirred for 15 min while the reaction temperature was maintained at 0°C. NaBF_4_ (165 mg, 1.5 mmol) in 0.5 mL of water was added dropwise to the reaction mixture at 0°C. After the mixture was stirred for 5 min, a 250 mL of water was added, and the reaction solution was stirred and refluxed at 120°C for 30 min. The reaction solution was adjusted to pH 12 with 2 M NaOH, and the aqueous mixture was extracted with ethyl acetate. The organic layer was dried over Na_2_SO_4_, and the solvent was removed *in vacuo*. The residue ((+)-Ves-OH, (+)-compound 6) was used in the next reaction without further purification.

NaNO_2_ (69 mg, 1 mmol) and I_2_ (254 mg, 1 mmol) were dissolved in 10 mL of 50% methanol. After being stirred for 30 min at room temperature, crude (+)-compound 6 (275 mg) in 1 mL of methanol was added dropwise to the reaction mixture while the reaction temperature was maintained at 0°C. After shaking the flask for 4 h at room temperature, the reaction solution was adjusted to pH 12 with 2-M NaOH, and the aqueous mixture was extracted with ethyl acetate. The organic layer was dried over Na_2_SO_4_, and the solvent was removed *in vacuo*. The residue was purified by chromatography on silica gel using chloroform/methanol (5:1) as the eluent to obtain (+)-compound 7 ((+)-IV-OH) (23.0 mg, 22% from (+)-compound 5) as a brown powder. At the same time, unreacted (+)-compound 6 ((+)-Ves-OH) (50.2 mg) was collected as a yellow powder.

(+)-Compound 6: ^1^H NMR (CDCl_3_) *δ* 1.17 to 1.34 (4H, m), 1.58 to 1.97 (8H, m), 2.14 (1H, m), 2.21 to 2.35 (2H, m), 2.39 to 2.49 (1H, m), 2.75 (2H, m), 2.96 (1H, d), 3.42 (1H, m), 5.30 (1H, s), 6.78 (2H, d), and 7.09 (2H, d); mass spectrum (MS, ESI) *m*/*z* 276 (M + H)^+^. (+)-Compound 7: ^1^H NMR (CDCl_3_) *δ* 1.13 to 1.34 (4H, m), 1.62 to 1.94 (8H, m), 2.01 (1H, m), 2.19 to 2.32 (2H, m), 2.39 (1H, m), 2.67 to 2.80 (2H, m), 2.93 (1H, m), 3.41 (1H, m), 5.30 (1H, s), 6.91 (1H, d), 7.08 (1H, d), and 7.50 (1H, s); MS (ESI) *m*/*z* 402 (M + H)^+^. Specific rotation is ${\left[\alpha \right]}_{D}^{22}=+23.5{}^{\circ}\left(c=0.0014g/\text{mL},\text{methanol}\right)$.

### Preparation of (−)-enantiomer of 4-[1-(2-hydroxycyclohexyl)piperidine-4-yl]-2-iodophenol ((−)-IV-OH)

(−)-IV-OH was synthesized in the same manner as was (+)-IV-OH using the (−)-enantiomer of vesamicol as a starting material instead of the (+)-enantiomer of vesamicol. ^1^H NMR (CDCl_3_) *δ* 1.23 (4H, m), 1.50 to 2.00 (8H, m), 2.09 (1H, m), 2.18 to 2.30 (2H, m), 2.40 (1H, m), 2.75 (2H, m), 2.96 (1H, d), 3.41 (1H, m), 5.30 (1H, s), 6.92 (1H, d), 7.08 (1H, d), and 7.55 (1H, s); mass spectrum (ESI) (*m*/*z*) 402 [M + H]^+^. Specific rotation is ${\left[\alpha \right]}_{D}^{22}=-22.9{}^{\circ}\left(c=0.0014g/\text{mL},\text{methanol}\right)$.

### *In vitro* competitive binding assay

Animal experimental protocols were approved by the Committee on Animal Experimentation of Kanazawa University. Experiments with animals were conducted in accordance with the Guidelines for the Care and Use of Laboratory Animals of Kanazawa University. The animals were housed with free access to food and water at 23°C with a 12-h alternating light/dark schedule. The rat brain and liver membranes for binding experiments were prepared from rat brains without cerebellum and rat liver in male Sprague–Dawley rats (200 g, Japan SLC, Inc., Hamamatsu, Japan), respectively, using a method described previously [21,26].

A sigma-1 receptor binding assay was performed using the following method. Rat cerebral membranes (465- to 1,193-μg protein) were incubated with 5-nM (+)-[^3^H]pentazocine and various concentrations of vesamicol analogs or sigma ligands (from 10^−10^ to 10^−5^ M) in 0.5 ml of 50 mM Tris–HCl (pH 7.8) for 90 min at 37°C. The incubated samples were quickly diluted with 5 mL of ice-cold Tris–HCl (pH 7.8) buffer followed by rapid filtration through Whatman Grade GF/B glass fiber filters (GE Healthcare UK Ltd., Amersham, UK) presoaked in 0.5% polyethylenimine using a cell harvester (Brandel, Gaithersburg, MD, USA). Filters were washed three times with 5 mL of ice-cold buffer. Nonspecific binding was determined in the presence of 10-μM (+)-pentazocine. Radioactivity retained on the filters was measured with a liquid scintillation counter (LSC-5100; Aloka, Tokyo, Japan).

A sigma-2 receptor binding assay was performed using the following method. Rat liver membranes (123- to 179-μg protein) were incubated with 5-nM [^3^H]DTG and each test compound (from 10^−10^ to 10^−5^ M) in 0.5 mL of 50-mM Tris–HCl (pH 7.8) for 90 min at 37°C in the presence of 1-μM (+)-pentazocine to mask sigma-1 sites. Nonspecific binding was determined in the presence of 10-μM DTG and 1-μM (+)-pentazocine. The incubated samples were treated in the same manner as described for the sigma-1 receptor binding assays.

### Preparation of (+)-[^125^I]IV-OH

(+)-^125^I]IV-OH was prepared by the chloramine-T method [27]. Briefly, ^125^I]sodium iodide solution (3.7 MBq/1 μL) was added to (+)-Ves-OH (6) in 100 μL of 0.1-M PBS pH 6.0 (10 mg/mL). Following mixing, 10 μL of chloramine-T aqueous solution (1 mg/mL) was added. After 10 min of standing at room temperature, the reaction mixture was quenched with 10 μL of Na_2_H_2_SO_5_ (0.72 mg/mL) and then purified by reversed phase (RP)-HPLC performed with a Cosmosil 5C_18_-MS-II column (4.6 × 150 mm; Nacalai Tesque, Kyoto, Japan) at a flow rate of 1 mL/min with a gradient mobile phase. Mobile phase A was water with 0.1% triethylamine; phase B was methanol with 0.1% triethylamine. The gradient conditions were as follows: 0 to 10 min, 70% to 80% B; 10 to 11 min, 80% to 100% B; and 11 to 20 min, 100% B. The column temperature was maintained at 40°C.

### Determination of the partition coefficient

The partition coefficient of (+)-^125^I]IV-OH was measured as described previously [23]. The partition coefficient was determined by calculating the ratio of counts per minute/milliliter in 1-octanol to that in the 0.02-M phosphate buffer and expressed as a common logarithm (log *P*).

### Cellular uptake experiments *in vitro*

Radiotracer uptake studies were performed in monolayer cultures of DU-145 prostate cancer cell lines, which were obtained from ATCC (Manassas, VA, USA). Cells were grown in cell culture dishes in RPMI 1640 medium with phenol red, 10% heat-inactivated fetal bovine serum (FBS), 100-μg/mL glutamine, 100-units/mL penicillin, and 100-μg/mL streptomycin at 37°C in a humidified atmosphere of 95% air and 5% carbon dioxide. Cells were plated on 6-well tissue culture plates (4 × 10^5^ cells/well) for 24 h before the study and incubated at 37°C in the culture medium without FBS containing (+)-^125^I]IV-OH or (+)-^125^I]*p*IV (3.7 kBq/well), which was prepared by a method of a previous study [23] for different time intervals (15, 30, 60, and 120 min). For the washout experiment, tumor cells exposed to a medium containing (+)-^125^I]IV-OH or (+)-^125^I]*p*IV for 60 min were washed with phosphate buffered saline (PBS) and incubated in fresh (nonradioactive) medium without FBS at 37°C for 15, 30, and 60 min. To investigate the inhibition of uptake with an excess of sigma ligand, the reduced uptake of (+)-^125^I]IV-OH or (+)-^125^I]*p*IV was also examined by incubation with 10 μM of haloperidol. After incubation, cells were washed twice with ice-cold PBS and resolved by adding 0.5 mL of 1-M NaOH. The solutions were then collected and the radioactivity was determined with an auto well gamma counter (ARC-380; Aloka) and corrected for background radiation. The radioactivity of each sample was normalized for the protein level, which was determined using a Protein Assay Bicinchoninate Kit (Nacalai Tesque).

### Biodistribution experiments of (+)-[^125^I]IV-OH and (+)-[^131^I]pIV in tumor-bearing mice

(+)-^131^I]*p*IV was prepared using a method described previously [23]. To produce tumors, approximately 5 × 10^6^ of the prepared DU-145 cells was injected subcutaneously into the right dorsum of 4-week-old male BALB/c nude mice (15 to 19 g, Japan SLC, Inc.). Biodistribution experiments were performed at approximately 14- to 21-day post-inoculation, i.e., when tumors reached a palpable size. Groups of four mice were intravenously administered with 100 μL of a mixed solution of (+)-^125^I]IV-OH (37 kBq) and (+)-^131^I]*p*IV (37 kBq). At 10-min, 1-, 3-, and 24-h post-injections, the mice were killed. Tissues of interest were removed and weighed, and radioactivity counts were determined with an auto well gamma counter and corrected for background radiation. A window from 16 to 71 keV was used for measuring ^125^I and one from 300 to 433 keV for ^131^I. The crossover of ^125^I activity into the ^131^I channel was negligible. Correlation factors to eliminate any crossover of ^131^I activity into ^125^I were determined by measuring the ^131^I standard in both windows.

### Blocking studies

For blocking studies, the above-mentioned DU-145 tumor-bearing mice were intravenously administered with 100 μL of (+)-^125^I]IV-OH (37 kBq) mixed with an excess of each unlabeled sigma ligand, haloperidol (10 μmol/kg), SA4503 (10 μmol/kg) [28], or (+)-*p*IV (10 μmol/kg). At 1-h post-injection, the mice were killed, and biodistribution experiments were conducted as described above.

### Metabolite analysis in blood, tumor, and other tissues

For metabolite analysis, the above-mentioned DU-145 tumor-bearing mice were intravenously administered with 100 μL of (+)-[^125^I]IV-OH (3.7 MBq). At 10-min and 1-h post-injections, the mice were killed. Blood was collected and tissues of interest were removed. The blood was centrifuged at 1,000×*g* for 10 min at 4°C. After the plasma was collected, an equivalent volume of acetonitrile/water mixture (1:1) was added to the plasma. The mixture was centrifuged at 1,000×*g* for 10 min at 4°C. The tissues of interest (0.2 to 0.5 g) were homogenized in 1 mL of acetonitrile-water mixture (1:1). Each homogenized sample was centrifuged at 1,000×*g* for 10 min at 4°C. The supernatants were analyzed by TLC with a chloroform/methanol mixture (5:1) as a developing solvent. TLC plates were exposed to phosphor imaging plates (BAS IP SR 2025 E, Fujifilm, Tokyo, Japan) for 48 h. The exposed imaging plates were evaluated using an imaging scanner (BAS 5000 Bio-Imaging Analyzer, Fujifilm).

### Statistical evaluation

A paired Student's *t* test was used for the biodistribution experiments. A one-way analysis of variance (ANOVA) followed by Dunnett's *post hoc* test compared to the control group was used for experiments in the blocking study. Results were considered statistically significant at *p* < 0.05.

## Results

### Preparation of (+)-IV-OH, (−)-IV-OH, and (+)-[^125^I]IV-OH

**Scheme 1:**
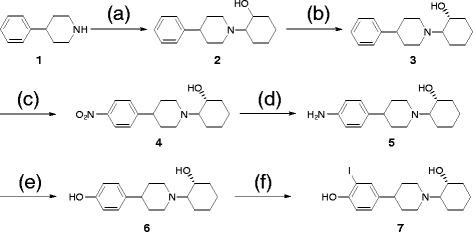
**Synthesis of (+)-IV-OH.** Reagents: (**a**) cyclohexene oxide, (**b**) (+)-di-*p*-toluoyl-D*-*tartaric acid, (**c**) HNO_3_, H_2_SO_4_, (**d**) Fe, HCl, (**e**) HCl, NaNO_2_, NaBF_4_, and (**f**) NaNO_2_, I_2_.

**Figure 2:**
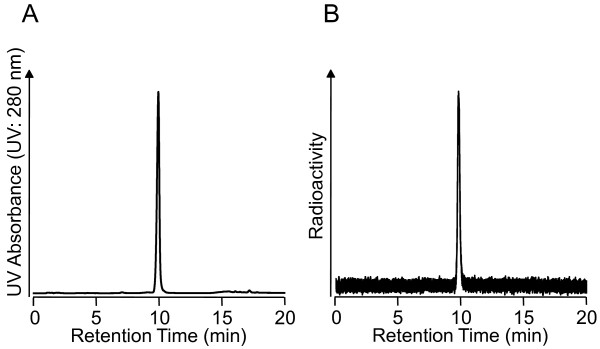
**RP-HPLC chromatograms of (A) nonradioactive (+)-IV-OH and (B) (+)-[**^**125**^**I] IV-OH after purification.** Condition: flow rate was 1 mL/min. Mobile phase A was water with 0.1% triethylamine, and phase B was methanol with 0.1% triethylamine. The gradient conditions are as follows: 0 to 10 min, 70% to 80% B; 10 to 11 min, 80% to 100% B; and 11 to 20 min, 100% B.

Syntheses of (+) and (−)-IV-OH are outlined in Scheme 1. (+)-Ves-OH (6) was prepared from (+)-compound 5 by diazotization and hydrolysis. Iodination was performed from (+)-Ves-OH (6) to obtain (+)-IV-OH (7). The overall yield of (+)-IV-OH was 8.5%. (+)-[^125^I]IV-OH was prepared by the chloramine-T method from (+)-Ves-OH (6) under no-carrier-added conditions with high radiochemical yield (69%). After purification by RP-HPLC, (+)-[^125^I]IV-OH showed radiochemical purities of over 98%. The specific activity of the no-carrier-added preparation must be comparable to that of [^125^I]NaI. The identity of (+)-[^125^I]IV-OH was verified by a comparison of retention time with the nonradioactive (+)-IV-OH (7) (Figure 2).

### *In vitro* competitive binding assay

Binding affinities of (+)-IV-OH, (−)-IV-OH, (+)-Ves-OH, (+)-vesamicol, haloperidol, and pentazocine to sigma receptors (sigma-1 and sigma-2) are shown in Table 1. The binding affinities of (+)-IV-OH to sigma-1 and sigma-2 were greater than those of (−)-IV-OH. (+)-IV-OH (*K*_i_ = 22.8 nM for sigma 1, *K*_i_ = 146.9 nM for sigma 2) showed the same degree of affinity for sigma receptors as (+)-vesamicol (*K*_i_ = 19.1 nM for sigma 1 and *K*_i_ = 159.3 nM for sigma 2), which is a mother compound, but (+)-IV-OH showed lesser affinity for the sigma 1 receptor than (+)-pentazocine (*K*_i_ = 10.0 nM) or haloperidol (*K*_i_ = 6.4 nM), which are known as sigma ligands.

**Table 1:** Affinities (nM) of IV-OH and reference compounds for sigma receptors

	**Sigma-1 (*K***_**i**_**)**	**Sigma-2 (*K***_**i**_**)**
(+)-IV-OH	22.8 (6.3)	146.9 (13.1)
(−)-IV-OH	45.5 (9.8)	165.5 (69.2)
(+)-Ves-OH	178.6 (25.2)	97.2 (3.1)
(+)-Vesamicol	19.1 (1.8)	159.3 (43.9)
Haloperidol	6.4 (0.7)	63.0 (7.2)
Pentazocine	10.0 (1.1)	2,417.7 (326.2)

*K*_i_ values derived from IC_50_ values according to the equation: *K*_i_ = IC_50_/(1 + *C*/*K*_d_), where *C* is the concentration of the radioligand, and each *K*_d_ is the dissociation constant of the corresponding radioligand ([^3^H]pentazocine to sigma-1 (*K*_d_ = 19.9 nM) and [^3^H]DTG to sigma-2 (*K*_d_ = 22.3 nM)). Values are means (SEM) of three or four experiments.

### Partition coefficient

Determination of the partition coefficient resulted in that the log *P* value of (+)-^125^I]IV-OH was 1.13 ± 0.01. This result indicates that the lipophilicity of (+)-^125^I]IV-OH is much less than that of (+)-^125^I]*p*IV, whose log *P* value is 2.08 [23].

### Cellular uptake experiments *in vitro*

**Figure 3:**
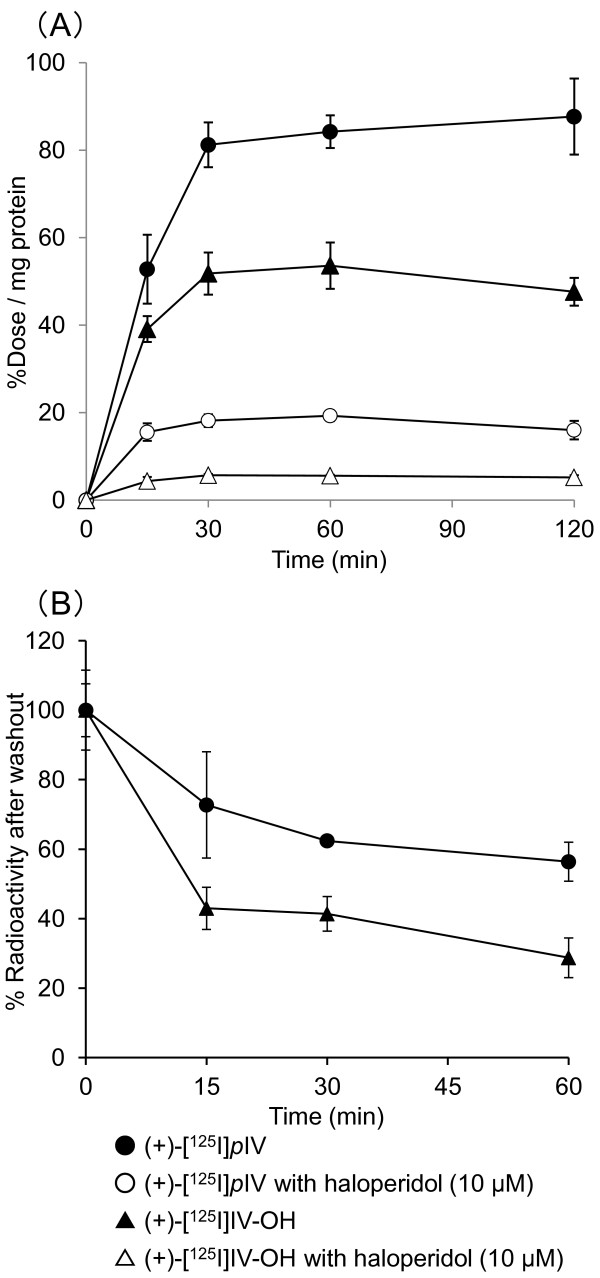
**Cell uptake and washout study.** (**A**) Time-dependent accumulation of (+)-[^125^I]IV-OH and (+)-[^125^I]*p*IV in DU-145 tumor cells with or without the addition of haloperidol (10 μM) into the medium. (**B**) Remaining percentage of radioactivity in cells after changing to fresh medium.

Cellular uptake experiments *in vitro* demonstrated a rapid uptake of (+)-[^125^I]IV-OH and (+)-[^125^I]*p*IV during the initial phase in DU-145 cells (Figure 3A). The uptakes of both radiotracers were saturated at 30 min; that of (+)-[^125^I]IV-OH was lower than that of (+)-[^125^I]*p*IV. The accumulation of (+)-[^125^I]IV-OH and (+)-[^125^I]*p*IV was remarkably lessened in the presence of a sigma ligand, haloperidol at 10 μM in culture medium (Figure 3A). Figure 3B shows the percentages of radioactivity of (+)-[^125^I]IV-OH and (+)-[^125^I]*p*IV in DU-145 cells to the time point of medium replacement in the washout experiment. In this case, the uptake of each radiotracer with haloperidol as a nonspecific uptake was subtracted from the uptake of each radiotracer to obtain a specific uptake via sigma receptor. The radioactivity of both radiotracers in the cells was released in a time-dependent manner after replacement of the medium. Approximately 71% of intracellular (+)-[^125^I]IV-OH was released into the supernatant from the DU-145 cells within 60-min after the medium replacement, while 44% of intracellular (+)-[^125^I]*p*IV had been released from the cells by that time.

### Biodistribution experiments of (+)-[^125^I]IV-OH and (+)-[^131^I]pIV in tumor-bearing mice

Table 2 lists the biodistribution of (+)-[^125^I]IV-OH and (+)-[^131^I]*p*IV in DU-145 tumor-bearing mice. (+)-[^125^I]IV-OH showed high uptake in tumor and low radioactivity levels in blood and muscle as well as (+)-[^131^I]*p*IV. (+)-[^131^I]*p*IV tended to be retained in most tissues, especially tumor. On the other hand, (+)-[^125^I]IV-OH was cleared from most tissue, including tumor, and almost no radioactivity was observed in any tissue at 24-h post-injection. In the liver, as we expected, the radioactivity levels of (+)-[^125^I]IV-OH were significantly lower at all time points compared with those of (+)-[^131^I]*p*IV. At the same time, the accumulation of (+)-[^125^I]IV-OH in the stomach was low, indicating that deiodination was not observed *in vivo*.

**Table 2:** **Biodistribution of radioactivity after concomitant intravenous injection of (+)-[**^**125**^**I]IV-OH and (+)-[**^**131**^**I]*p*IV in tumor-bearing mice**

**Analog**	**Tissue**	**Time of post-injection**			
		**10 min**	**1 h**	**3 h**	**24 h**
(+)-[^125^I] IV-OH	Blood	1.28 (0.13)*	1.04 (0.10)*	0.43 (0.09)**	0.00 (0.00)*
	Tumor	9.55 (0.64)**	5.45 (0.70)	2.57 (0.41)	0.06 (0.04)**
	Liver	7.82 (0.32)*	9.80 (0.94)**	2.81 (0.33)*	0.06 (0.03)*
	Kidney	35.01 (2.83)*	15.30 (1.80)	4.89 (0.87)**	0.04 (0.01)*
	Intestine	5.26 (0.61)*	7.96 (0.72)*	4.84 (0.95)	0.14 (0.02)*
	Spleen	8.11 (1.24)	1.84 (0.11)*	0.60 (0.12)*	0.01 (0.02)*
	Pancreas	25.04 (2.40)*	12.80 (0.97)*	2.94 (0.49)*	0.01 (0.01)*
	Lung	9.74 (1.59)*	1.85 (0.18)*	0.63 (0.11)*	0.01 (0.02)*
	Heart	3.57 (0.40)*	0.78 (0.05)*	0.27(0.00)**	0.01 (0.01)*
	Stomach^a^	1.18 (0.36)	0.62 (0.18)	0.55 (0.25)	0.03 (0.02)*
	Brain	5.14 (0.50)*	1.04 (0.08)*	0.22 (0.02)*	0.00 (0.00)*
	Muscle	2.20 (0.58)	0.45 (0.03)*	0.26 (0.10)	0.01 (0.01)*
(+)-[^131^I]*p*IV	Blood	0.45 (0.03)	0.28 (0.02)	0.17 (0.01)	0.50 (0.01)
	Tumor	7.20 (0.40)	5.79 (0.37)	6.46 (3.42)	6.81 (2.60)
	Liver	9.83 (0.39)	13.31 (1.25)	13.05 (0.46)	10.28 (0.59)
	Kidney	18.47 (1.59)	11.72 (1.90)	7.37 (0.89)	6.15 (0.30)
	Intestine	4.31 (0.78)	5.14 (0.12)	3.20 (0.13)	2.59 (0.24)
	Spleen	8.12 (1.09)	8.99 (1.00)	4.04 (0.30)	2.52 (0.23)
	Pancreas	16.58 (1.82)	21.94 (2.50)	27.35 (0.24)	27.36 (0.70)
	Lung	26.10 (3.39)	13.13 (3.28)	6.59 (0.56)	3.01 (0.46)
	Heart	8.66 (0.81)	5.55 (0.70)	2.11 (0.37)	0.96 (0.07)
	Stomach^a^	1.07 (0.32)	0.73 (0.26)	0.62 (0.19)	0.49 (0.06)
	Brain	6.02 (0.74)	6.47 (0.73)	4.12 (0.30)	2.22 (0.10)
	Muscle	2.03 (0.53)	1.62 (0.22)	0.72 (0.29)	0.42 (0.03)

Data are expressed as percent injected dose per gram tissue. Each value represents the mean (SD) for three or four animals. ^a^Data are expressed as percent injected dose. Significance was determined using paired Student's *t* test (*p < 0.01 vs. (+)-[^131^I]*p*IV, **p < 0.05).

### Blocking studies

**Figure 4:**
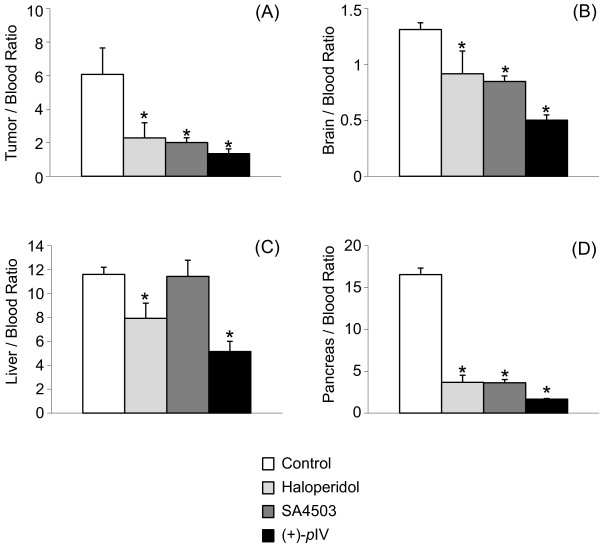
**Comparison of tumor/tissue uptake ratio (mean ± SD) of (+)-[**^**125**^**I]IV-OH.** At 1-h post-injection under no-carrier-added condition and under co-injection of haloperidol, SA4503, or (+)-*p*IV. Significance was determined using one-way ANOVA followed by Dunnett's *post hoc* test (*p* < 0.01 vs. control (asterisk)).

The effects of some sigma ligands on tumor uptake of (+)-[^125^I]IV-OH at 1-h post-injection are shown as the ratios of percent injected dose per gram of the tumor (A), the brain (B), the liver (C), or the pancreas (D) as tissues which have highly abundant sigma receptor density to blood in Figure 4. In this case, the radioactivity level in the blood was changed by co-injection of sigma ligands. Thus, the figures are shown as tissue/blood ratio. Co-injection of an excess amount of haloperidol, SA4503, or (+)-*p*IV, which are sigma ligands, resulted in a significant decrease in the uptake ratios of tumor to blood, brain to blood, and pancreas to blood after injection of (+)-[^125^I]IV-OH.

### Metabolite analysis in blood, tumor, and other tissues

**Figure 5:**
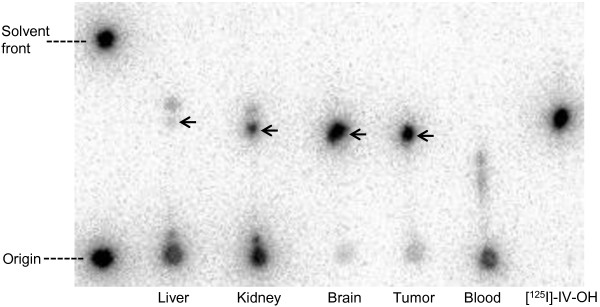
**Representative radio-TLC image of metabolites at 10-min post-injection of (+)-[**^**125**^**I]IV-OH in tumor-bearing mice.** TLC was performed with chloroform/methanol (5:1) as the mobile phase. Arrows indicate the spots of intact (+)-[^125^I]IV-OH.

Table 3 and Figure 5 show the results of metabolite analyses after intravenous injection of (+)-[^125^I]IV-OH in DU-145 tumor-bearing mice. The proportions of the intact form in the tumor and brain were much higher than were those in the blood, liver, and kidney. In the blood, liver, and kidney, almost no intact (+)-[^125^I]IV-OH was observed at 1-h post-injection (0.9%, 0.1%, and 2.6%, respectively).

**Table 3:** **Analysis of metabolites after intravenous injection of (+)-[**^**125**^**I]IV-OH in tumor-bearing mice**

**Tissue**	**Time of post-injection**	
	**10 min**	**1 h**
Blood	17.0 (9.0)	0.9 (1.0)
Tumor	93.1 (4.3)	70.2 (5.6)
Liver	5.4 (7.4)	0.1 (0.2)
Kidney	19.8 (3.7)	2.6 (1.8)
Brain	96.4 (1.1)	73.7 (8.4)

Data are expressed as percent of intact (+)-[^125^I]IV-OH. Each value represents the mean (SD) for three samples.

## Discussion

In competitive binding assays of sigma receptors, it was reported that vesamicol and iodovesamicol analogs enantioselectively bound to the sigma-1 receptor [21]. Namely, the (+)-enantiomers of the vesamicol analogs have higher affinities than the (−)-enantiomers of the vesamicol analogs. In this study, IV-OH, as well as the previous vesamicol analogs, enantioselectively bound to the sigma-1 receptor. The binding affinity of (+)-IV-OH to sigma-1 receptor was greater than that of (−)-IV-OH (Table 1). Meanwhile, (+)-Ves-OH (6) showed much less affinity for sigma-1 compared to (+)-vesamicol. Accordingly, the introduction of a hydroxyl group at the para-position of the benzene ring in vesamicol markedly reduced the affinity for sigma-1 receptor. By introduction of iodine to (+)-Ves-OH, (+)-IV-OH showed a much higher affinity for sigma-1 receptor compared to that of (+)-Ves-OH. However, the reduction of the affinity for sigma-1 receptor by the introduction of the hydroxyl group was large. The affinity of (+)-IV-OH (*K*_i_ = 22.8 nM) for sigma-1 receptor was same degree as that of (+)-vesamicol (*K*_i_ = 19.1 nM), which is a parent compound, and was less than that of (+)-*p*IV (*K*_i_ = 1.30 nM [22]).

A lower log *P* value of (+)-^125^I]IV-OH (1.13 ± 0.01) by the introduction of the hydroxyl group compared with that of (+)-^125^I]*p*IV (2.08 ± 0.02 [23]) was expected to improve biodistribution of radioiodinated vesamicol analogs as imaging agents. The value was less than we expected because the calculated log *P* value for IV-OH and *p*IV were 4.06 and 4.45, respectively, using CS ChemDraw Ultra software (Cambridge Soft Corporation, Cambridge, MA, USA). Certainly, the accumulation of radioactivity in the liver does not depend only on the physicochemical characteristics. It is known that sigma receptors are highly expressed in the liver [29]. Although the accumulation of radioactivity in the liver should be partly related to the affinities for sigma receptors, in this study, the lower hepatic uptake and the higher renal uptake of (+)-^125^I]IV-OH at early time points after injection compared with those of (+)-^131^I]*p*IV in the biodistribution experiments (Table 2) could be partly explained because of the lower lipophilicity of (+)-^125^I]IV-OH. The suggestion is consistent with the previous reports that decreasing the lipophilicity of radiolabeled compounds resulted in lower hepatic uptake [30].

In the biodistribution experiments in tumor-bearing mice, (+)-[^125^I]IV-OH showed higher radioactivity uptake in the DU-145 tumor than we expected. Actually, at 10-min post-injection, (+)-[^125^I]IV-OH showed significantly higher uptake than (+)-[^131^I]*p*IV although the affinities for the sigma receptors of (+)-IV-OH were lower than were those of (+)-*p*IV. The exact causes of the high uptake (+)-[^125^I]IV-OH in tumor are not clear, but one cause could be because of the higher radioactivity of (+)-[^125^I]IV-OH in blood than that of (+)-[^131^I]*p*IV at 10-min post-injection. Moreover, we assumed that the high uptake of (+)-[^125^I]IV-OH in tumor may also be derived from other mechanisms except via sigma receptors. However, in the cellular uptake study with the DU-145 cells *in vitro*, the cellular uptake of (+)-[^125^I]IV-OH was remarkably inhibited in the presence of haloperidol (10 μM) in the culture medium (Figure 3A). In the blocking study, the co-injection with excess amounts of sigma ligands significantly decreased the tumor/blood uptake ratio of radioactivity (Figure 4A). These results indicate that the high uptake of (+)-[^125^I]IV-OH in the DU-145 tumor is mainly caused via sigma receptors. In the uptake study with the DU-145 cells *in vitro*, the specific uptake, which is defined by subtracting uptake with haloperidol as non-specific uptake, of (+)-[^125^I]IV-OH at 15-min after incubation was almost the same as that of (+)-[^125^I]*p*IV (Figure 3A). The result also supports the high tumor uptake of (+)-[^125^I]IV-OH in the biodistribution experiments.

Meanwhile, (+)-^125^I]IV-OH cleared faster from the tissues, and almost no radioactivity was observed in any tissue at 24-h post-injection while (+)-^131^I]*p*IV tended to remain in most tissues. This could be partly because of the difference in metabolism rates. In the blood, liver, and kidney, almost no intact (+)-^125^I]IV-OH was observed at 1-h post-injection (Table 3). On the other hand, large proportions of radioactivity existed in an intact form in almost all tissues except blood at 1-h post-injection of (+)-^125^I]*p*IV [23]. Accordingly, the metabolism of (+)-^125^I]IV-OH should be faster than that of (+)-^131^I]*p*IV. Furthermore, the difference of the clearance rate from tissues between (+)-^125^I]IV-OH and (+)-^131^I]*p*IV might be partly because of the difference of the affinity for sigma receptors. Namely, the faster clearance of (+)-^125^I]IV-OH might be partly from the lower affinity of (+)-^125^I]IV-OH for sigma receptors than that of (+)-^131^I]*p*IV. Actually, in the washout experiments in the cell uptake study, more (+)-^125^I]IV-OH was released from the DU-145 cells compared to that of (+)-^125^I]*p*IV after changing to fresh media (Figure 4B).

In the *in vitro* binding assay, (+)-IV-OH preferred the sigma-1 subtype, but the selectivity was not so high (approximately 6.4-fold). (+)-IV-OH may bind to not only the sigma-1 receptor but also to the sigma-2 receptor in tumor because the sigma-1 and sigma-2 receptors are highly expressed on DU-145 cells [31]. In the blocking study, the decrease of tumor/blood uptake ratios of (+)-^125^I]IV-OH between haloperidol and SA4503 were almost the same (Figure 4A, SA4503 binds mainly to sigma-1, and haloperidol is a nonselective sigma ligand [32].) Therefore, the results suggest that (+)-^125^I]IV-OH ought to mainly bind to the sigma-1 receptor. Meanwhile, in the blocking study for the liver, SA4503 did not inhibit liver uptake of (+)-^125^I]IV-OH (Figure 4C). However, this result should not be simply accepted. Accumulations of radiotracers in the liver with co-injection of blocking compounds were affected by not only the affinities for receptors but also the physiochemical characteristics of the tracer and the competitive inhibition of uptake or metabolism by the blocking compounds, etc. In fact, it was reported that the uptake of radioactivity in the liver after injection of a radiolabeled sigma-1 ligand with high affinity was not reduced by blocking study using haloperidol [33].

## Conclusions

In conclusion, these results indicate that (+)-IV-OH has potential as a sigma receptor imaging agent because of its high tumor uptake via sigma receptor, lower hepatic uptake, and faster clearance from the tissues in tumor-bearing mice compared to that of radioiodinated (+)-*p*IV.

## Competing interests

The authors declare that they have no competing interests.

## Authors’ contributions

KO designed and took part in all aspects of this study and drafted the manuscript. HK carried out all experiments and analyzed the data of *in vitro* binding assay and animal experiments. KS has contributed in the concept and design of the study and participated in the synthesis of a precursor of (+)-[^131^I]*p*IV. YK contributed in the *in vitro* binding assay and metabolite analysis. TiK, ToK, and AO contributed in the synthesis of (+)-[^125^I]IV-OH and in analyzing and interpreting the data. All authors read and approved the final manuscript.
